# ST-Segment Elevation in a Patient With Vocal Cord Cancer: A Great Mimicker

**DOI:** 10.1016/j.jaccas.2026.108452

**Published:** 2026-05-21

**Authors:** Luis Rene Puglla-Sanchez, Javier Mario Valcuende Rosique, David Mendez Portuburu, Pablo Revilla Marti

**Affiliations:** Department of Cardiology, Clinico Lozano Blesa University Hospital, Zaragoza, Spain

**Keywords:** cardiac metastasis, pseudoinfarction pattern, squamous cell carcinoma

## Abstract

**Case Summary:**

Cardiac metastasis mimicking ST-segment elevation myocardial infarction is a rare “pseudoinfarction pattern.” A 58-year-old man with recurrent laryngeal carcinoma presented with pleuritic chest pain and anterolateral ST-segment elevation. High-sensitivity troponin levels plateaued (70-76 ng/L). Emergent coronary angiography was deferred due to the pain's quality, the absence of dynamic electrocardiogram evolution, and bedside echocardiographic identification of a right ventricular mass. Positron emission tomography-computed tomography confirmed extensive neoplastic infiltration. This pattern results from a persistent injury current at the tumor-myocardium interface. Recognition is vital to avoid futile invasive procedures and guide appropriate palliative systemic therapy.

**Take-Home Messages:**

Persistent ST-segment elevation in an oncology patient without typical dynamic evolution should raise suspicion of myocardial tumor infiltration. Multimodality imaging, particularly emergent bedside echocardiography, is essential to differentiate malignant “injury currents” from acute coronary syndromes and avoid unnecessary invasive interventions.

## Graphical Abstract

**Figure:**
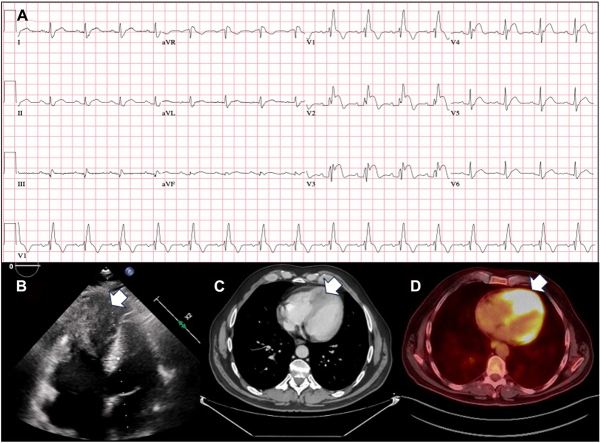

## Case Summary

A 58-year-old man with recurrent laryngeal squamous cell carcinoma (SCC) presented with a 6-hour history of pleuritic chest pain and diaphoresis. He was tachycardic (99 beats/min) and hypotensive (90/60 mm Hg). Past oncologic treatment included induction chemotherapy with cisplatin and docetaxel (3 cycles) and concurrent chemoradiotherapy, which was completed 4 months before presentation.

**Figure 1:**
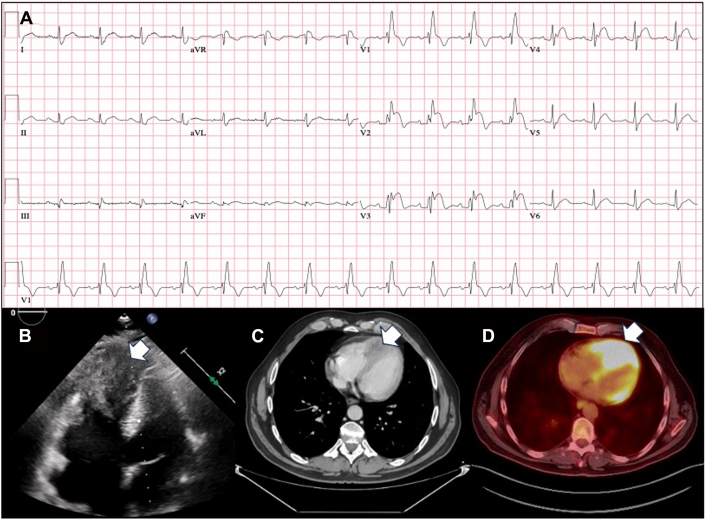
Electrocardiographic and Imaging Findings (A) ECG showing a right bundle branch block and prominent anterolateral ST-segment elevation. (B) Apical 4-chamber echocardiography revealing a large RV mass (arrow). (C) Contrast-enhanced CT showing an infiltrative mass involving the RV and pericardium (arrow). (D) Positron emission tomography-CT demonstrating intense fluorodeoxyglucose avidity in the RV lateral wall and apex (arrow). Note that the extensive neoplastic infiltration of the RV apical and lateral walls directly correlates with the prominent anterolateral ST-segment elevation pattern observed on the admission ECG. CT = computed tomography; ECG = electrocardiogram; RV = right ventricle.

An initial electrocardiogram (ECG) (Figure 1A) showed a right bundle branch block and prominent ST-segment elevation (STE) in V_1_-V_5_ and I-aVL. High-sensitivity troponin T levels showed a plateau pattern (76, 70, and 75 ng/L), and the chest pain remained unresponsive to low-dose vasodilators. Although current clinical guidelines recommend emergent coronary angiography as the default strategy for typical STE myocardial infarction presentations, the procedure was deferred in this instance. This decision weighed the atypical pleuritic quality of the chest pain and the absence of classical dynamic ECG evolution (such as Q-wave formation or T-wave inversion) against the immediate diagnostic clarification provided by emergent bedside transthoracic echocardiography, which identified a large infiltrative right ventricular (RV) mass (Figure 1B).

Subsequent computed tomography and 18-F-fluorodeoxyglucose positron emission tomography-computed tomography confirmed a 38-mm hilar lung nodule and intense hypermetabolism in the RV lateral wall and apex, infiltrating the pericardium (Figures 1C and 1D). After the diagnosis of stage IV SCC with cardiac metastasis, management shifted to palliative intent. The patient initiated systemic therapy (carboplatin-paclitaxel-pembrolizumab) with good immediate tolerance. No cardiac-directed interventions (radiotherapy or surgery) were performed due to the extent of systemic disease.

The ECG differential diagnosis was broad. Typical anterolateral STE myocardial infarction was considered but lacked reciprocal changes and dynamic evolution. Acute myopericarditis usually presents with diffuse concave STE- and PR-segment depression, which were absent. Acute pulmonary embolism with RV strain typically manifests as an S1Q3T3 pattern or T-wave inversions in V_1_-V_4_, rather than localized, profound STE.

This “pseudoinfarction pattern” is driven by a persistent injury current at the tumor-myocardium interface. Mechanistic series established that this phenomenon arises from a localized voltage gradient created by the direct replacement of healthy myocytes with neoplastic cells, peritumoral inflammation, and alterations in ionic conductance, specifically potassium efflux, within zones of tumor necrosis. Unlike the transient injury of an acute coronary occlusion, neoplastic infiltration provides a static substrate for a permanent electrical potential difference, which explains the characteristic lack of typical T-wave or Q-wave evolution noted in more recent reviews. A nearly identical phenomenon was recently described by Chunta et al^1^; however, our case is further confounded by a baseline right bundle branch block and highlights the primary role of bedside transthoracic echocardiography over cardiac magnetic resonance for rapid triage in unstable patients. Although cardiac magnetic resonance is the gold standard for tissue characterization, it was deferred here due to the patient's clinical instability and the immediate transition to a palliative trajectory.

Cardiac metastasis in head-and-neck SCC frequently involves right-sided chambers and carries an extremely poor prognosis, with short-term survival being the norm.^2^ This case illustrates that in oncology patients, especially those with a history of cardiotoxic treatments such as platinum-based therapy and radiation, persistent STE requires a broad differential diagnosis. Although these treatments can cause direct myocardial or pericardial injury, persistent elevation without dynamic evolution should strongly raise suspicion of metastatic infiltration. Early multimodality imaging is essential to differentiate malignant injury currents from acute coronary syndromes and guide appropriate palliative goals.^3^

## Funding Support and Author Disclosures

The authors have reported that they have no relationships relevant to the contents of this paper to disclose.

**Take-Home Messages**

• Persistent ST-segment elevation in an oncology patient without typical dynamic evolution should raise suspicion of myocardial tumor infiltration.
• Multimodality imaging, particularly emergent bedside echocardiography, is essential to differentiate malignant "injury currents" from acute coronary syndromes and avoid unnecessary invasive interventions.
